# Preoperative Prostatic Artery Embolization Combined With Transurethral Resection of the Prostate Versus Transurethral Resection of the Prostate Alone in Large Benign Prostatic Hyperplasia: A Systematic Review and Meta-Analysis

**DOI:** 10.7759/cureus.111326

**Published:** 2026-06-22

**Authors:** Hasan Alchalabi, Omar Amro, Rafie Ebrahimi Ghaei, Conall Murphy, Muhammad Ali

**Affiliations:** 1 Surgery, Royal Victoria Hospital, Belfast, GBR; 2 Internal Medicine, Aneurin Bevan University Health Board, Cwmbran, GBR

**Keywords:** benign prostatic hyperplasia, embolization techniques, interventional radiology-guided embolization, lower urinary tract symptoms, meta-analysis, minimally invasive procedures, prostatic artery embolization, transurethral resection of prostate

## Abstract

Benign prostatic hyperplasia (BPH) with large prostate volume presents substantial surgical challenges for transurethral resection of the prostate (TURP), encompassing heightened intraoperative haemorrhage risk, prolonged operative duration, and increased perioperative morbidity. Prostatic artery embolization (PAE) has been investigated as a minimally invasive adjunct to reduce prostatic vascularity prior to TURP, potentially optimising perioperative outcomes. This systematic review and meta-analysis evaluated the comparative efficacy and safety of PAE combined with TURP (PAE+TURP) versus TURP alone in patients with large-volume BPH.

PubMed (MEDLINE), Embase, Scopus, and Web of Science were searched from inception through January 2026. Randomised controlled trials (RCTs) and cohort studies comparing PAE+TURP with TURP alone in large-volume BPH were eligible. The primary outcome was the International Prostate Symptom Score (IPSS). Secondary outcomes comprised operative time, maximum urinary flow rate (Qmax), and quality of life (QoL). Pooled mean differences (MDs) with 95% confidence intervals (CI) were derived using random-effects models.

Three studies (one RCT and two cohort studies) totalling 261 patients (88 PAE+TURP, 173 TURP alone) were included. PAE+TURP yielded significantly greater IPSS improvement than TURP alone (MD=-2.07; 95% CI: -3.15 to -0.99; p<0.001; I²=0%). Subgroup analyses stratified by follow-up duration demonstrated no significant between-group difference in studies reporting three-month outcomes, whereas studies reporting six-month outcomes favoured PAE+TURP. Operative time was significantly shorter with PAE+TURP (MD=-38.7 minutes; 95% CI: -66.1 to -11.3; p=0.01), albeit with substantial heterogeneity (I²=97.8%). Qmax and QoL did not differ significantly between groups (both p=0.08). The single RCT was rated low risk of bias using the Cochrane Collaboration Risk of Bias 2 (RoB 2) tool; both retrospective cohort studies received a good-quality rating on the Newcastle-Ottawa Scale, though residual confounding inherent to observational designs cannot be excluded.

Preoperative PAE followed by TURP was associated with shorter operative time and superior lower urinary tract symptom (LUTS) control compared with TURP alone in large-volume BPH. The limited evidence base and methodological heterogeneity across current studies underscore the need for large-scale randomised trials to establish long-term benefits and standardise patient selection criteria.

## Introduction and background

Benign prostatic hyperplasia (BPH) is a highly prevalent histological condition among ageing men, with autopsy series reporting tissue changes in nearly 90% of men by their ninth decade [1]. Clinically, glandular enlargement obstructs the bladder outlet and gives rise to lower urinary tract symptoms (LUTS) including nocturia, urgency, reduced stream calibre, and incomplete bladder emptying [1,2]. Inadequately managed BPH carries the risk of progressive sequelae such as recurrent haematuria, urinary tract infections, secondary bladder dysfunction, and upper tract deterioration [1]. When pharmacological therapy proves insufficient, operative intervention is required [3,4].

Transurethral resection of the prostate (TURP) is the benchmark surgical procedure for BPH, reliably improving symptoms and urinary flow with well-established long-term durability [3,4]. Its limitations become pronounced when the prostate exceeds 80-100 mL in volume: resection of such large glands demands prolonged operative time, exposes patients to greater intraoperative blood loss and fluid absorption, heightens perioperative morbidity, and risks incomplete tissue removal [1,5]. Alternative approaches, including open simple prostatectomy, holmium laser enucleation of the prostate (HoLEP), and, more recently, Aquablation, have been used for larger prostates and may address some of these limitations, although each carries distinct technical requirements, costs, learning curves, and complication profiles [6].

Prostatic artery embolization (PAE) achieves symptom relief through the selective occlusion of prostatic arterial supply, inducing ischaemia-mediated glandular atrophy and progressive volume reduction [7,8]. As a standalone modality, PAE delivers sustained LUTS improvement, though its benefit accrues more gradually than surgery and may be incomplete in very large glands [9,10]. Consequently, interest has grown in deploying PAE preoperatively before TURP, with the premise that prior arterial devascularisation curtails intraoperative haemorrhage, enhances endoscopic field clarity, and enables more efficient and thorough resection, which is particularly valuable when the prostate is markedly enlarged [1,5,11].

The combined strategy has been examined in several recent publications. Tang et al. demonstrated that PAE+TURP in glands exceeding 100 mL significantly curtailed operative duration, intraoperative blood loss, and postoperative catheter dependency compared with TURP alone, with superior International Prostate Symptom Score (IPSS), quality of life (QoL), and prostate volume outcomes at 12 months [5]. Zhiyu et al. reported a large retrospective cohort study in giant-gland BPH (≥100 mL) confirming that the combined approach conferred better two-year LUTS and flow outcomes while complication severity distributions remained equivalent [1]. Lee et al. subsequently reported the first randomised controlled trial (RCT) comparing these strategies in prostates exceeding 80 mL, demonstrating a significant attenuation of perioperative haemoglobin decline and transfusion need with preoperative PAE, alongside equivalent resection efficiency and complication rates [11].

Taken together, these data suggest that PAE+TURP may mitigate the principal limitations of TURP monotherapy in large-gland BPH. However, heterogeneity across study designs, patient selection thresholds, embolization protocols, and outcome reporting has prevented firm conclusions regarding the superiority of the combined strategy. We therefore conducted this systematic review and meta-analysis to rigorously appraise the comparative efficacy and safety of preoperative PAE combined with TURP versus TURP alone in patients with large-volume BPH.

## Review

Methods

This systematic review and meta-analysis was conducted in accordance with the Cochrane Handbook for Systematic Reviews of Interventions [12] and the Preferred Reporting Items for Systematic Reviews and Meta-Analyses (PRISMA) 2020 statement [13].

Data Sources and Search Strategy

Electronic databases including PubMed (MEDLINE), Embase, Scopus, and Web of Science (WOS) were interrogated from database inception through January 2026. The search strategy combined the following terms: (“benign prostatic hyperplasia” OR “benign prostatic enlargement” OR BPH OR “prostatic hyperplasia”) AND (“transurethral resection of prostate” OR “transurethral resection of the prostate” OR TURP OR “transurethral prostatectomy” OR “prostate resection”) AND (“Therapeutic Embolization” OR “prostatic artery embolization” OR PAE). Neither language restrictions nor date filters were imposed.

Eligibility Criteria and Study Outcomes

RCTs and cohort studies were considered eligible when they fulfilled the following criteria within a Population, Intervention, Comparison, and Outcome (PICO) framework: (1) adult male participants aged ≥18 years without restriction on demographic characteristics, (2) direct comparison of functional outcomes between PAE+TURP and TURP alone in patients with large-volume BPH, and (3) quantitative reporting of at least one primary or secondary outcome of interest. Exclusion criteria comprised the following: age below 18 years, absence of primary outcome data, and publication types including reviews, editorials, correspondence, conference abstracts without full datasets, and animal studies. IPSS was the prespecified primary outcome. Secondary outcomes were maximum urinary flow rate (Qmax), QoL, and operative time.

Study Selection

Duplicate records were removed using EndNote (Clarivate, London, United Kingdom) [14]. The de-duplicated records were then transferred to Rayyan (Rayyan Systems Inc., Cambridge, Massachusetts, United States) for blinded independent screening [15]. Two reviewers independently screened titles and abstracts, followed by full-text appraisal of potentially eligible records. Disagreements were adjudicated by discussion or, where consensus was unattainable, by a third reviewer. Reference lists of all included studies were forward- and backward-tracked to capture any studies not retrieved by the primary search.

Data Extraction

Two reviewers independently extracted data using a pre-piloted Microsoft Excel form (Microsoft Corporation, Redmond, Washington, United States). Domains captured included the following: (1) study-level characteristics (design, country, sample size, inclusion criteria, follow-up duration, and principal conclusions), (2) participant-level baseline data (age, body mass index (BMI), prostate volume, serum prostate-specific antigen (PSA), and pre-treatment outcome values), and (3) post-treatment outcomes (IPSS, QoL, Qmax, and operative time). A third reviewer audited all extracted fields before analysis, with residual discrepancies resolved by consensus.

Risk-of-Bias Assessment

Two reviewers independently appraised methodological quality using study design-specific instruments. For the RCT, the Cochrane Collaboration Risk of Bias 2 (RoB 2) tool was employed [16], covering five domains: randomisation process, deviation from intended interventions, missing outcome data, outcome measurement, and selection of reported results. Each domain received a "low risk", "some concerns", or "high risk" designation. Cohort studies were appraised using the Newcastle-Ottawa Scale (NOS) [17], examining three domains: cohort selection, comparability, and outcome assessment. Studies were classified as good, moderate, or poor quality according to the cumulative NOS score. Discordant assessments were resolved through discussion or third-party adjudication.

Statistical Analysis

Continuous outcomes were synthesised as mean differences (MD) with 95% confidence intervals (CI) using the DerSimonian-Laird random-effects model. A two-tailed p-value threshold of 0.05 was used to define statistical significance. Given the variation in follow-up intervals across the included studies, outcome data from each study's final reported time point were used as the primary analysis. A pre-planned subgroup analysis stratified by follow-up duration was undertaken to explore differences in reported outcomes across studies with different follow-up periods. Heterogeneity was quantified using Cochran's Q test and the I² statistic; values of p<0.1 or I²≥50% were considered indicative of substantial heterogeneity [12]. Random-effects meta-regression was performed to explore associations between study-level baseline covariates (mean age, prostate volume, and PSA) and pooled outcomes. All analyses were conducted in Stata MP version 18 for Windows (StataCorp LLC, College Station, Texas, United States).

Results

Patient Characteristics

**Figure 1:**
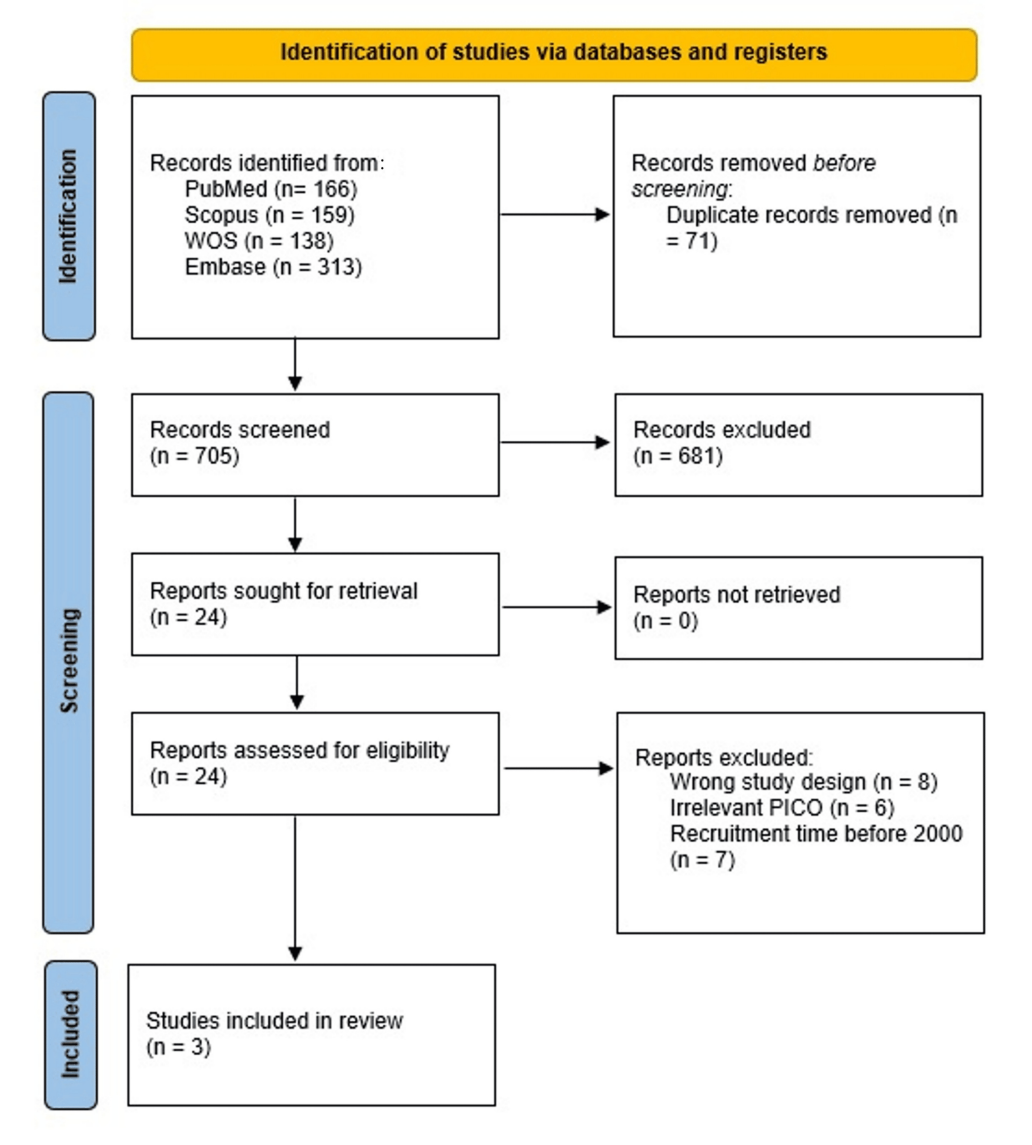
The PRISMA flow diagram PRISMA: Preferred Reporting Items for Systematic Reviews and Meta-Analyses; WOS: Web of Science; PICO: Population, Intervention, Comparison, and Outcome

Database searching identified 776 records in total. After de-duplication (n=71 removed), 705 records proceeded to title and abstract screening. Full-text review was performed for 24 records, of which three met the eligibility criteria and were included in the final analysis [1,5,11]. The PRISMA 2020 flow diagram is shown in Figure 1.

The three studies originated from China and Singapore, collectively enrolling 261 male participants: 88 in the PAE+TURP arm and 173 in the TURP-alone arm. Follow-up ranged from three to 24 months. The study characteristics are detailed in Table 1. The mean participant age was 73.4±7.96 years (PAE+TURP: 74.5±8.88 years; TURP alone: 72.9±7.41 years).

**Table 1:** Summary of the included studies BPH: benign prostatic hyperplasia; PAE: prostatic artery embolization; RCT: randomised controlled trial; TURP: transurethral resection of the prostate

Study ID	Total sample size (N)	Study design	Country	Population description	Follow-up	Conclusion
Zhiyu et al. (2023) [1]	211	Retrospective analysis	China	Patients with giant (>100 mL) BPH treated with PAE+TURP or TURP alone	24 months	In contrast to TURP alone, PAE+TURP is more expensive but provides better postoperative outcomes; there is no significant difference in terms of the severity grade distribution of postoperative complications
Tang et al. (2021) [5]	30	Retrospective analysis	China	Patients with large (>100 mL) BPH who underwent TURP and PAE+TURP	12 months	Compared to TURP alone, PAE+TURP should be promoted, because of its greater efficacy and safety in treating large BPH and fewer post-surgical complications
Lee et al. (2025) [11]	20	RCT	Singapore	Patients with prostate volume >80 mL with indications for TURP	3 months	Preoperative PAE reduces intraoperative blood loss in men with large prostates undergoing TURP but did not impact resection efficiency or complication rate

The baseline characteristics are presented in Table 2.

**Table 2:** Baseline characteristics of the included studies' patients All variables are represented with mean and standard deviation. IPSS: International Prostate Symptom Score; Qmax: maximum urinary flow rate; PVR: post-void residual urine volume; PSA: prostate-specific antigen; PAE: prostatic artery embolization; RCT: randomised controlled trial; TURP: transurethral resection of the prostate; QoL: quality of life

Study ID	Group	Sample size	Age (years)	Prostate volume (mL)	Serum PSA (ng/mL)	IPSS	QoL	Qmax (mL/s)	PVR (mL)
Lee et al. (2025) [11]	PAE+TURP	10	72.93 (10.92)	126.67 (45.58)	15.77 (13.16)	14.67 (12.9)	3.67 (1.72)	13.7 (5.59)	NA
	TURP alone	10	70.1 (8.86)	131 (47.3)	11.03 (11.18)	23.3 (12.9)	3.67 (4.3)	6.4 (3.87)	NA
Zhiyu et al. (2023) [1]	PAE+TURP	61	73.49 (8.48)	123.60 (29.29)	6.95 (3.53)	26.95 (4.55)	5.00 (0.77)	5.63 (2.91)	166.5 (125.00)
	TURP alone	150	72.47 (7.12)	123.50 (25.32)	7.03 (3.35)	26.53 (3.87)	4.95 (0.61)	6.28 (2.80)	143.5 (7.97)
Tang et al. (2021) [5]	PAE+TURP	17	79.12 (7.97)	163.57 (52.97)	5.48 (1.12)	33.06 (1.95)	5.65 (0.49)	5.44 (1.67)	117.18 (5.93)
	TURP alone	13	79.46 (6.63)	151.02 (30.95)	5.18 (1.02)	33.46 (1.33)	5.69 (0.48)	5.66 (1.25)	117.31 (3.33)

Risk-of-Bias Assessment

**Figure 2:**
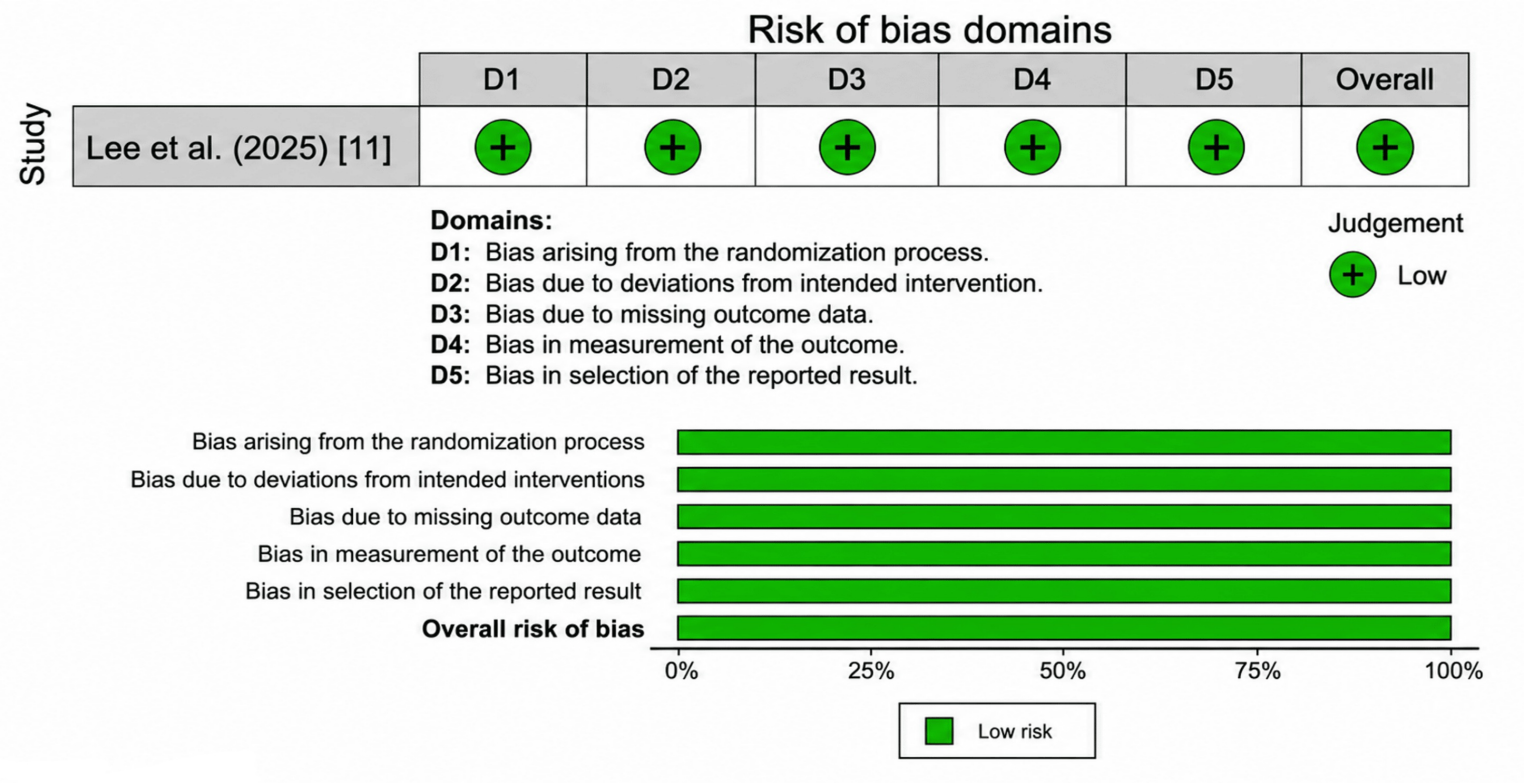
RoB 2 assessment Risk-of-bias assessment for the included randomised controlled trial using the RoB 2 tool [16]: D1, bias from the randomisation process; D2, bias from deviations from intended interventions; D3, bias from missing outcome data; D4, bias in outcome measurement; and D5, bias in selection of reported results. Lee et al. (2025) [11] has a low overall risk of bias. RoB 2: Cochrane Collaboration Risk of Bias 2

RoB 2 assessment [16] indicated that Lee et al. [11] carried a low overall risk of bias across all five evaluated domains (Figure 2).

NOS appraisal [17] of both cohort studies (Zhiyu et al. [1] and Tang et al. [5]) returned a good-quality rating for selection, comparability, and outcome domains (Table 3).

**Table 3:** NOS for the quality assessment of observational studies Assessed using the NOS [17]. Stars represent quality indicators within each domain. NOS: Newcastle-Ottawa Scale

Study	Selection	Comparability	Outcome	Overall score	Overall judgment
Zhiyu et al. (2023) [1]	★★★	★★	★★★	★★★★★★★★	Good quality
Tang et al. (2021) [5]	★★★	★★	★★	★★★★★★★	Good quality

Primary Outcome: IPSS

**Figure 3:**
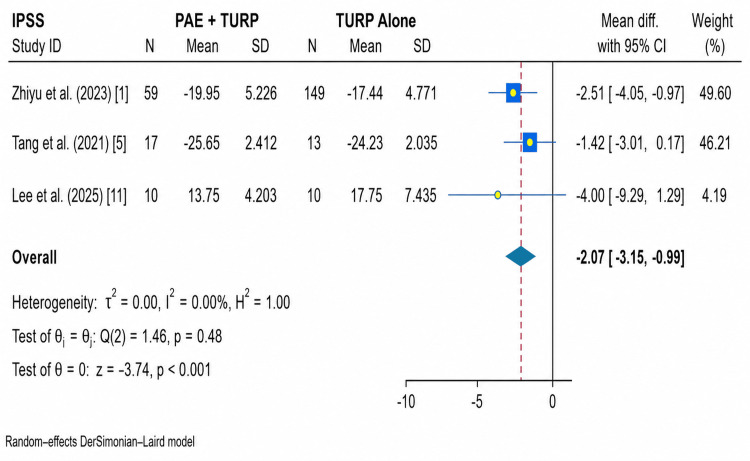
Forest plot of IPSS Forest plot of pooled MD in IPSS between PAE+TURP and TURP alone groups. IPSS: International Prostate Symptom Score; PAE: prostatic artery embolization; TURP: transurethral resection of the prostate; MD: mean difference; CI: confidence interval

IPSS data from all 261 participants (88 PAE+TURP; 173 TURP alone) were pooled. Post-treatment IPSS was significantly lower in the PAE+TURP group (MD=-2.07; 95% CI: -3.15 to -0.99; p<0.001; I²=0%; Figure 3). Although I²=0% was observed, this finding should be interpreted cautiously given that heterogeneity statistics have limited statistical power when only three studies are included. Consequently, the absence of detected heterogeneity should not be interpreted as definitive evidence of homogeneity.

**Figure 4:**
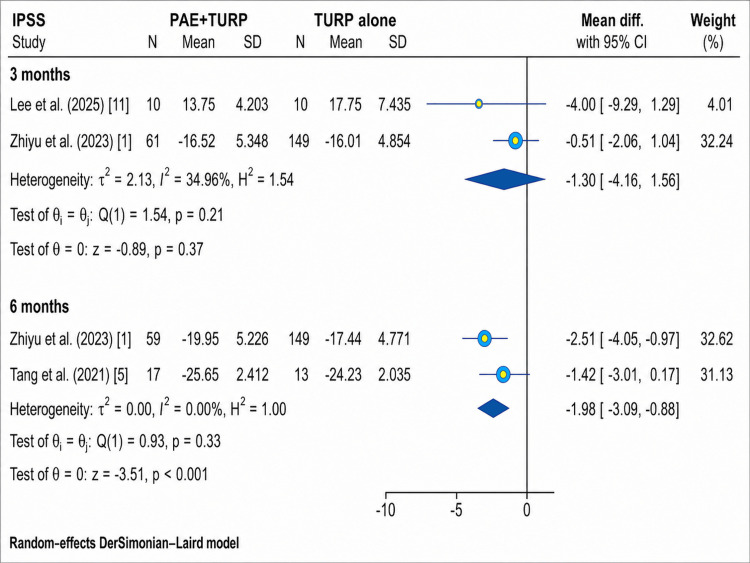
Forest plot of subgroup analysis of IPSS Forest plot of subgroup analysis of IPSS stratified by follow-up duration (3 vs. 6 months). IPSS: International Prostate Symptom Score; PAE: prostatic artery embolization; TURP: transurethral resection of the prostate; MD: mean difference; CI: confidence interval

Subgroup analyses were conducted according to follow-up duration. At the three-month follow-up, no statistically significant between-group difference was apparent (MD=-1.3; 95% CI: -4.16 to 1.56; p=0.37; I²=34.96%). Among studies reporting the six-month outcomes, a statistically significant difference favouring PAE+TURP was observed (MD=-1.98; 95% CI: -3.09 to -0.88; p<0.001; I²=0%; Figure 4).

Secondary Outcomes

**Figure 5:**
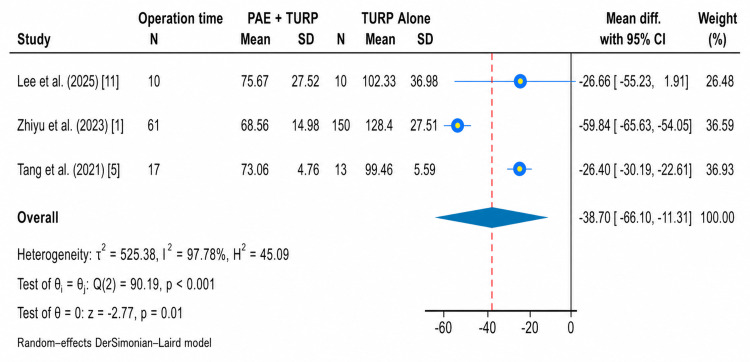
Forest plot of operation time Forest plot of pooled MD in operative time (minutes). Substantial heterogeneity observed (I²=97.78%). PAE: prostatic artery embolization; TURP: transurethral resection of the prostate; MD: mean difference; CI: confidence interval

Operative time: Operative time was significantly shorter with PAE+TURP than with TURP alone (MD=-38.7 minutes; 95% CI: -66.1 to -11.3; p=0.01; I²=97.78%; Figure 5). The high heterogeneity indicates that the magnitude of time-saving varied substantially across centres, likely attributable to differences in operator experience, embolic agent selection, embolization technique, the PAE-to-TURP interval, and baseline prostate dimensions.

**Figure 6:**
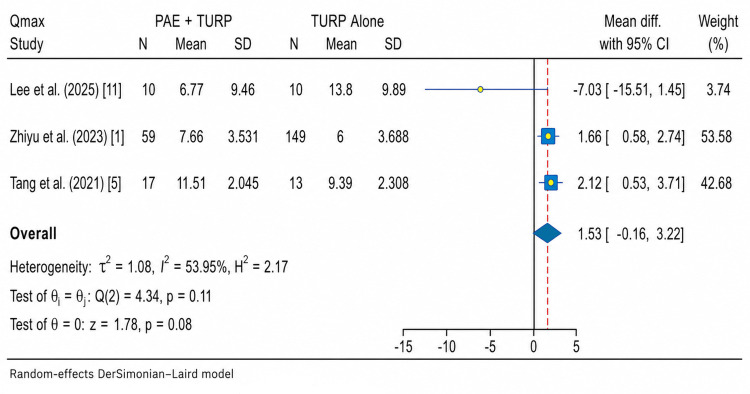
Forest plot of Qmax Forest plot of pooled MD in Qmax (mL/s). Qmax: maximum urinary flow rate; PAE: prostatic artery embolization; TURP: transurethral resection of the prostate; MD: mean difference; CI: confidence interval

Qmax and QoL: Pooled Qmax did not differ significantly between groups (MD=1.53; 95% CI: -0.16 to 3.22; p=0.08; I²=53.95%; Figure 6).

**Figure 7:**
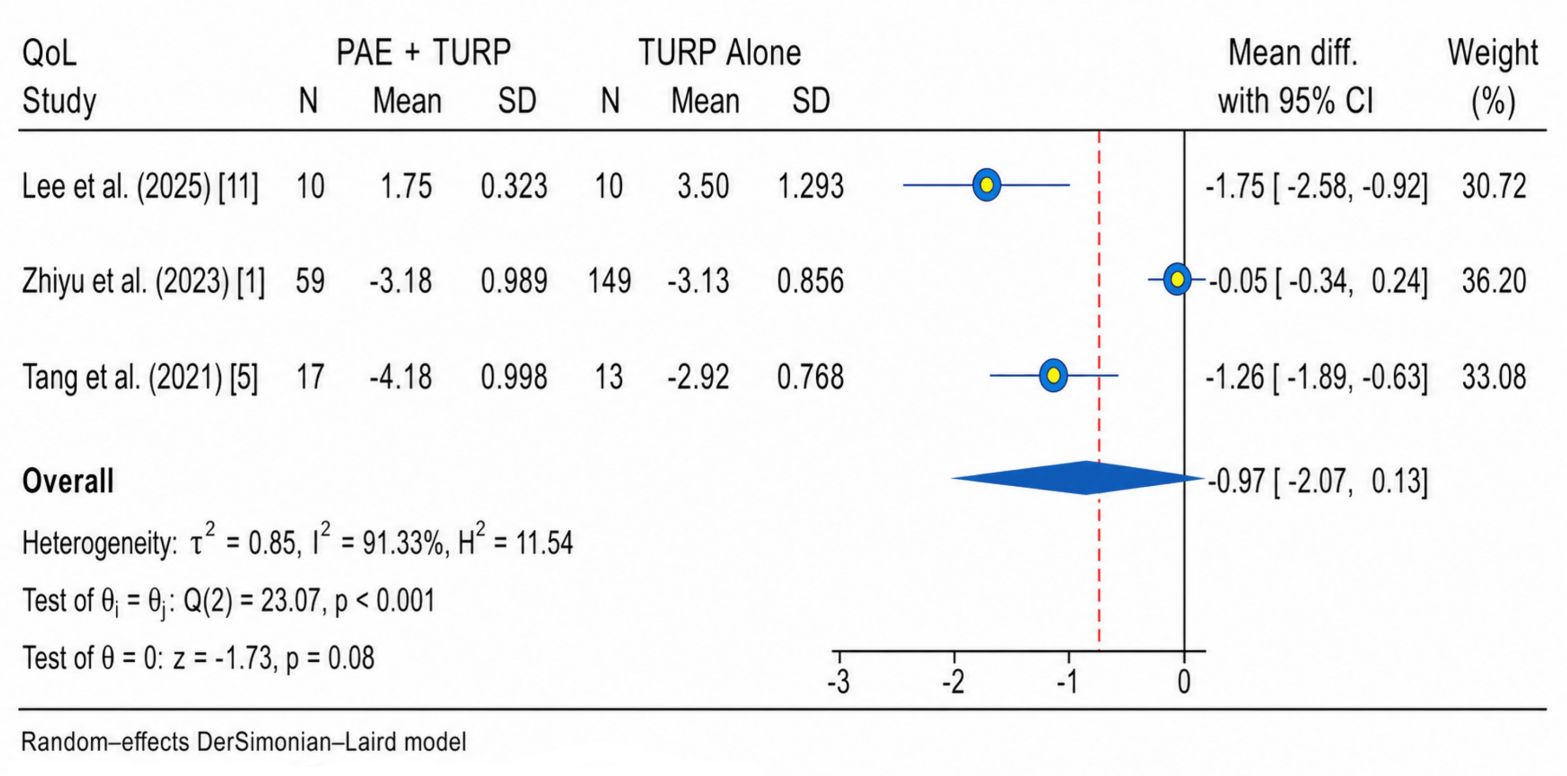
Forest plot of QoL Forest plot of pooled MD in QoL score. QoL: quality of life; PAE: prostatic artery embolization; TURP: transurethral resection of the prostate; MD: mean difference; CI: confidence interval

QoL scores similarly showed no statistically significant between-group difference (MD=-0.97; 95% CI: -2.07 to 0.13; p=0.08; I²=91.33%; Figure 7). The elevated heterogeneity for QoL most likely reflects variation in measurement instruments and patient case-mix across studies.

Discussion

To our knowledge, this is the first meta-analytic synthesis specifically comparing combined preoperative PAE followed by TURP against TURP monotherapy in the setting of large-volume BPH (prostate ≥80-100 mL). Three studies comprising 261 participants were analysed, including one RCT and two retrospective cohort studies. PAE+TURP was associated with significantly greater IPSS reduction and meaningfully shorter operative times relative to TURP alone, while Qmax and QoL outcomes did not reach statistical significance.

Study Outcomes and Mechanisms

The pooled IPSS reduction favouring PAE+TURP (MD=-2.07) suggests superior symptom relief with the combined strategy. The six-month subgroup analysis (MD=-1.98) demonstrated a statistically significant difference favouring PAE+TURP, whereas the three-month estimate derived from a single small RCT did not reach statistical significance. However, these subgroup findings reflect differences between studies rather than longitudinal follow-up of the same patient cohort and should therefore be interpreted with caution. Although the studies reporting longer follow-up demonstrated favourable IPSS outcomes with PAE+TURP, the limited number of included studies and differences in study design, patient populations, and follow-up duration preclude firm conclusions regarding the temporal evolution of treatment effect.

The approximately 38.7-minute reduction in operative time with PAE+TURP suggests a procedural advantage of the combined approach in patients with large-volume BPH. However, the substantial heterogeneity observed for operative time (I²≈98%) suggests that the magnitude of benefit varied considerably across institutions, potentially reflecting differences in operator expertise, embolization technique, embolic agents, procedural timing, and baseline prostate size. Prospective trials should mandate explicit reporting of these variables.

The non-significant findings for Qmax and QoL may stem from several sources: limited statistical power due to the small cumulative sample size and the short three-month follow-up of the only RCT; the dominant influence of TURP itself on urodynamic recovery, to which PAE may contribute only incrementally; or inconsistency in QoL instrument selection and administration across studies. The very high QoL heterogeneity (I²≈91%) underscores these measurement disparities.

Relation to Previous Literature

The individual studies comprising this review have each reported directionally consistent benefits. Zhiyu et al. documented that PAE+TURP attenuated intraoperative haemorrhage and improved two-year LUTS outcomes in giant-gland BPH [1], while Tang et al. observed fewer postoperative complications and superior 12-month IPSS scores with the combined strategy [5]. The first RCT by Lee et al. corroborated a meaningful attenuation of perioperative haemoglobin decline and transfusion requirement, with equivalent resection efficiency [11].

The broader PAE evidence base provides important context. Standalone PAE has been shown to achieve significant and durable improvements in IPSS, Qmax, QoL, and prostate volume in multiple series and meta-analyses [7,8]. Head-to-head comparisons confirm that TURP produces faster and more pronounced urodynamic recovery than PAE alone, but at the cost of higher rates of ejaculatory dysfunction, urethral stricture, and longer inpatient admission [9,10].

Altman et al. conducted a systematic review and meta-analysis comparing PAE against a range of surgical and minimally invasive procedures for BPH, confirming the superiority of surgical approaches, including TURP, for short-term urodynamic outcomes, tempered by a higher adverse event burden [18]. The cumulative evidence supports further investigation of PAE+TURP as a treatment strategy for patients with large-volume BPH.

Current American Urological Association (AUA) guidelines affirm TURP as the reference standard surgical intervention for BPH refractory to pharmacotherapy, particularly in moderate-to-large prostates [19]. The mounting interest in PAE+TURP is underpinned by PAE's capacity to reduce prostatic arterial inflow before endoscopic surgery, moderate the inflammatory response, and induce gradual glandular atrophy, potentially reducing operative demands and accelerating postoperative recovery.

Study Limitations

Several methodological constraints warrant acknowledgement. The evidence base comprised only three studies, which severely restricted statistical power for secondary outcomes. Heterogeneity in institutional settings, embolic agent selection, embolization technique, the interval between PAE and TURP, and outcome reporting methodology limited generalisability. Variation in the definition of "large prostate" (thresholds of >80 mL and >100 mL), together with differences in baseline PSA and comorbidity profiles across studies, complicated direct comparisons. The sole RCT had a follow-up of only three months, precluding the assessment of medium- and long-term outcomes. Potential selective reporting of rare adverse events, quantitative haemorrhage data, catheterisation duration, and hospital length of stay represents a further limitation. These findings should be interpreted with caution when considering application to patients with significant comorbidities, anticoagulation requirements, or advanced frailty.

Furthermore, the evidence base consisted of only three studies, limiting the ability of heterogeneity statistics to detect true between-study variability and restricting confidence in subgroup analyses.

Implications for Clinical Practice

The present analysis suggests that PAE+TURP may yield clinically meaningful gains in large-gland BPH through improved LUTS control and a shorter operative footprint. The findings of this review are most applicable to patients with large-volume BPH (prostate volume >80-100 mL), reflecting the populations included in the available studies. Given the current evidence base and the dual procedural expertise required, PAE+TURP should currently be viewed as a specialist centre option rather than a standard-of-care pathway, pending confirmation from larger randomised studies.

Recommendations for Future Research

Adequately powered, multicentre RCTs with prespecified standardised embolization protocols (agent selection, technique, PAE-to-TURP interval) and uniform outcome definitions are essential to validate and extend these preliminary findings. Future studies should capture quantitative haemorrhage metrics, transfusion rates, catheterisation duration, and hospital length of stay in addition to patient-reported outcomes including erectile and ejaculatory function, overall satisfaction, and health-related QoL. Formal cost-effectiveness analyses comparing PAE+TURP with TURP alone and with HoLEP are needed to position this combined approach appropriately within current BPH management algorithms.

## Conclusions

This systematic review and meta-analysis provides evidence that combining preoperative PAE with TURP reduces operative time and achieves superior LUTS improvement compared with TURP monotherapy in patients with large-volume BPH. Subgroup analyses suggested a statistically significant benefit in studies reporting six-month outcomes; however, these findings reflect cross-study comparisons and should be interpreted cautiously. Nonetheless, the small evidence base, predominance of observational studies, and substantial methodological heterogeneity necessitate cautious interpretation. Robust, large-scale RCTs with extended follow-up and standardised protocols are required before PAE+TURP can be incorporated as a routine strategy within the BPH surgical management algorithm.
